# Diversity Measures in Environmental Sequences Are Highly Dependent on Alignment Quality—Data from ITS and New LSU Primers Targeting Basidiomycetes

**DOI:** 10.1371/journal.pone.0032139

**Published:** 2012-02-21

**Authors:** Dirk Krüger, Danuta Kapturska, Christiane Fischer, Rolf Daniel, Tesfaye Wubet

**Affiliations:** 1 Department of Soil Ecology, UFZ - Helmholtz Centre for Environmental Research, Halle (Saale), Germany; 2 Environmental Biotechnology, International Graduate School of Zittau, Zittau, Germany; 3 Department of Genomic and Applied Microbiology, Georg-August University of Göttingen and Institute of Microbiology and Genetics, Göttingen Genomics Laboratory, Göttingen, Germany; University of California Riverside, United States of America

## Abstract

The ribosomal DNA comprised of the ITS1-5.8S-ITS2 regions is widely used as a fungal marker in molecular ecology and systematics but cannot be aligned with confidence across genetically distant taxa. In order to study the diversity of Agaricomycotina in forest soils, we designed primers targeting the more alignable 28S (LSU) gene, which should be more useful for phylogenetic analyses of the detected taxa. This paper compares the performance of the established ITS1F/4B primer pair, which targets basidiomycetes, to that of two new pairs. Key factors in the comparison were the diversity covered, off-target amplification, rarefaction at different Operational Taxonomic Unit (OTU) cutoff levels, sensitivity of the method used to process the alignment to missing data and insecure positional homology, and the congruence of monophyletic clades with OTU assignments and BLAST-derived OTU names. The ITS primer pair yielded no off-target amplification but also exhibited the least fidelity to the expected phylogenetic groups. The LSU primers give complementary pictures of diversity, but were more sensitive to modifications of the alignment such as the removal of difficult-to align stretches. The LSU primers also yielded greater numbers of singletons but also had a greater tendency to produce OTUs containing sequences from a wider variety of species as judged by BLAST similarity. We introduced some new parameters to describe alignment heterogeneity based on Shannon entropy and the extent and contents of the OTUs in a phylogenetic tree space. Our results suggest that ITS should not be used when calculating phylogenetic trees from genetically distant sequences obtained from environmental DNA extractions and that it is inadvisable to define OTUs on the basis of very heterogeneous alignments.

## Introduction

A large proportion of the world's biogeochemically important terrestrial microorganisms are Fungi. Most of the symbiotic and saprobic fungal taxa that degrade plant-derived carbon compounds (e.g. lignin and cellulose) in forest ecosystems belong to the subphylum Agaricomycotina [1], [2]. Recent developments in extremely high-throughput sequencing technologies have made it more feasible to unravel their diversity on a large scale. To most fully survey the diversity of fungi in the environment, the choice of sequencing locus is extremely important, even if more than one locus will result in non-overlapping datasets that have to be evaluated separately.

In general, a sequencing target for phylogenetic reconstruction and for identifying different groups should be orthologous, alignable, and not saturated in the mutations that contain the phylogenetic signal. The extent to which some of the most widely used fungal marker genes satisfy above criteria is debatable. Phylogenetic analyses are tightly connected with alignment, as can also be seen in the development of tools that simultaneously optimize alignment and phylogenies such as POY and SATé-II [3], [4] and attempts to denoise phylogenomic and metagenomic datasets [5]. The internal transcribed spacer (ITS) region of the nuclear ribosomal DNA has been the locus of choice for analyzing phylogenetic relationships, especially at lower taxonomic ranks where variability may still allow unambiguous alignment. This is because of its large copy number (and thus high template availability) and the relative ease of designing both broad and selective PCR primers for it [6], [7], [8]. However, it should be noted that different species have different ITS copy numbers and that the copy number may be dependent on fungus' life stage at the time of sampling. A large amount of ITS sequence data is currently available in rapidly expanding public databases. To illustrate this growth, a keyword search for “ITS and Fungi and RNA” in the NCBI GenBank database conducted on December 1, 2010 yielded ca. 33 160 sequences; a search using the same keywords performed on December 20, 2011 yielded ca. 1 699 660 sequences. The ITS region was one of the loci used in the large AFTOL study on fungal evolution [9], [10] (along with some other ribosomal DNA (rDNA) and protein-coding sequences), although the only section of the ITS region considered during the phylogenetic analyses was the 5.8S rRNA gene [11]. It has been used extensively in molecular microbial ecology studies [1], [12]–[Bibr pone.0032139-Hanson1] [14] and was proposed as a fungal marker for the Barcode of Life [15], [16]. However, there are some caveats regarding its use in molecular ecology. Some of these also affect other rDNA loci, such as their highly repetitive nature (which may entail differences between gene copies [17], [18], [19]), the potential for primer bias that would result in some fungal groups' markers being amplified to a much greater extent than others [20], and the uncertainty regarding rDNA copy number variation between and within taxa, strains, and life stages [21]–[Bibr pone.0032139-Srikantha1] [Bibr pone.0032139-Rooney1] [Bibr pone.0032139-Ganley1] [25]. All of these factors complicate the use of the ITS for molecular quantification of fungi in the wild. Another aspect that limits phylogenetic accuracy is our lack of knowledge on the precise secondary structure of the pre-rRNA transcripts. The rRNA secondary structure constrains the evolution of rDNA because nucleotide sites evolve non-independently, with compensatory base changes. Consequently, they are unlikely to behave in line with the assumptions made in widely-used models of sequence evolution [26]–[Bibr pone.0032139-Higgs1] [Bibr pone.0032139-Smith1] [Bibr pone.0032139-Yu1] [29]. There are also some problems that affect the ITS more than alternative rDNA loci such as the 18S (SSU) or the 28S (LSU) genes. The ITS spacers (ITS1, ITS2) are highly variable in length and nucleotide sequence content [25], [30] and these fast-evolving sequences make alignment ambiguous and prone to noise. This makes them useful for taxonomy at lower ranks and for community fingerprinting methods [6], [31]–[Bibr pone.0032139-Zinger1] [Bibr pone.0032139-Robinson1] [34], but also makes them less desirable for methods in which consistent amplicon lengths are preferred, e.g. gradient gel fingerprinting [35], [36]. ITS-derived cDNA has been used to take snapshots of growing, metabolically active fungi [37], but this approach has not been widely tested.

Previous studies on fungal diversity in environmental samples have already focused on the LSU gene (e.g. [38]–[Bibr pone.0032139-Pivato1] [Bibr pone.0032139-Porter1] [41]), which does not suffer from some of the aforementioned shortcomings of the ITS.

Compared to the ITS region, the bulk of the LSU sequence exhibits a much lower degree of variability across large taxonomic distances, with the exception of its hypervariable D1–D12 core domains. The considerable length heterogeneity of these domains in eukaryotes [42]–[Bibr pone.0032139-BenAli1] [Bibr pone.0032139-Gillespie1] [Bibr pone.0032139-Ki1] [46] reduces the resolution near the tips of phylogenetic trees constructed using LSU sequence data relative to that achieved using different protein-coding sequences [47]. However, the use of such alternative sequences would require more extensive primer optimization to achieve selectivity for specific fungal groups in molecular ecology studies. In addition, the international sequence databases contain far fewer LSU reference sequences suitable for identifying query sequences than is the case for the ITS region.

There have been no concerted efforts to develop fine-tuned primers for selective amplification of the LSU gene from the higher taxonomic ranks of the Basidiomycota. In order to compare the phylogenetic resolution of the widely used ITS1F/4B primer pair [48] to that achievable for the LSU rDNA fragment, we designed primers that primarily amplify ca. 800 bp long LSU rDNA fragments from the Agaricomycotina (especially the Agaricales, but also the Boletales and the Russulales), which account for much of the fungal diversity in temperate European hardwood forests.

The study described in this paper had two main objectives: a) to design selective primers for amplifying typical Agaricomycotina LSU sequences and compare the potential of ITS and LSU rDNA fragments for assessing fungal diversity in environmental samples; and b) to establish simple pre-phylogenetic data exploration methods based on information entropy and the distance covered by the sequences within an Operational Taxonomic Unit (OTU) that will enable the practising molecular ecologist or mycologist to select appropriate primers and analyses for specific purposes.

## Materials and Methods

### Primer design

Oligonucleotide primers were designed to amplify the larger subunit (LSU) of the ribosomal DNA (28S rDNA) of the Agaricomycotina, with particular emphasis on targeting members of the order Agaricales. To cover the sequence variation within these fungi, reference sequences representing distinct species found in temperate forests were retrieved from the NCBI GenBank database (5 Agaricaceae: AY207233, AY635772, AY635775, DQ457685, DQ911601; 6 Bolbitiaceae: AY129384, AY207138, AY207178, AY207265, AY691807, DQ071696; 1 Cortinariaceae AF261524; 1 Entolomataceae AY207197; 2 Lycoperdaceae: AF261485, DQ071709; 2 Marasmiaceae: AY635776, DQ071718; 1 Omphalotaceae DQ470816; 8 Strophariaceae: AF261518, AY129382, AY207277, AY207310, AY380409, DQ071689, DQ674808, EF051055, and 8 Tricholomataceae: AF261328, AF261465, AF291305, AY207163, AY207230, AY647208, AY745709, DQ071713). These sequences were aligned using the ClustalX v. 2.0.12 program [49] and the alignments were kept in BioEdit v. 7.0.9.0 [50].

New primers were designed to amplify LSU fragments of ca. 800 bp using GeneFisher2 [51] and FastPCR v. 4.0.27 [52]. The Oligo Toolkit and Plotter (http://www.operon.com/tools/oligo-analysis-tool.aspx) was used to select primers and pairs of primers with the lowest possible tendency to undergo dimerisation or hairpin formation and with similar melting temperatures to ensure efficient annealing. Subsequently, NCBI-BLAST (BLASTn) [53] at the DDBJ website was used to check the specificity of the primers; the first 10,000 hits were considered, and the search was conducted against the entire database save for entries corresponding to environmental samples. In total, six primer candidates were tested against the DDBJ database and cultures, as outlined below. We ultimately settled on two primer pairs: Mix5 and Mix7 (Table 1). Mix5 was designed using GeneFisher and consists of primers nuLSU-Ag-0187-5′ and nuLSU-Ag-1003-3′. Mix7 was designed with FastPCR and consists of nuLSU-Ag-0176-5′ and nuLSU-Ag-1006-3′. Both selected primer pairs were used to amplify DNA extracted from soil samples. We also amplified ITS sequences in the soil sample DNA using the basidiomycete-specific internal transcribed spacer primer pair ITS1F (forward primer, complementary to the 3′ end of the SSU gene) and ITS4B (reverse primer, complementary to the 5′ section of the LSU gene) [48], [54]. The annealing temperatures of the candidate primer pairs were optimized by performing PCR on some extractions in a range of temperatures that included 1°C, 3°C, 5°C, 7°C and 9°C below the lower of the two calculated melting temperatures [55]. For convenience, the names ‘Mix5’ and ‘Mix7’ are used throughout the remainder of this paper when discussing the corresponding primer pairs; the individual primers belonging to each pair are listed in Table 1.

**Table 1:** Characteristics of primers and primer pairs.

Primer	Type*	Target region	Sequence 5′ to 3′	Primer pair	Tm(°C)**	Self hybridization	Primer dimer	Cycling conditons	Discardedsequences***	Off-target sequences***
nuLSU-Ag-0187-5′	f	LSU	AAGTCTCCTGGAATGGAGCGTCA	Mix5	64.55	2 4 bp-dimers	1 4 bp-dimer	initial denaturation 94°C for 10 min (1 cycle); 94°C for 1 min, 58°C for 1 min, and 72°C for 1 min (35 cycles) and final elongation 72°C for 10 min (1 cycle)	low quality 11.26%; insufficient length 3.85%; mismatches 0.10%	non-Agaricomycotina fungal misamplifications 0.20%
nuLSU-Ag-1003-3′	r	LSU	TTCTGCTATCCTGAGGGAAACTTC		62.86	-				
nuLSU-Ag-0176-5′	f	LSU	GGMCCGTGTRHAAGTYTCCTGG	Mix7	66.09	3 4 bp-dimers, 1 6 bp-dimer	2 4 bp-dimers		low quality 6.45%; insufficient length 3.67%; mismatches 0.36%	non-fungal misamplifications (plant) 0.27%
nuLSU-Ag-1006-3′	r	LSU	TGAGTTTCTGCTATCCTGAGGGAA		62.86	-				
ITS1F	f	ITS	CTTGGTCATTTAGAGGAAGTAA	ITS	57.08	-	1 4 bp-dimer	94°C for 10 min (1 cycle); 94°C for 1 min, 60°C for 1 min, and 72°C for 1 min ( 35 cycles); and 72°C for 10 min (1 cycle)	low quality 1.94%; insufficient length 0.53%-	-
ITS4B	r	ITS	CAGGAGACTTGTACACGGTCCAG		66.33	1 6 bp-dimer				

*f = forward, r = reverse;

**melting temperatures (Tm) of oligonucleotide sequences were calculated using Oligo Analysis Tool (http://www.operon.com/tools/oligo-analysis-tool.aspx),

***percentage values estimated from the total cloning/sequencing approach adopted in other biodiversity studies on sampled forest soils (ITS: 567, Mix5: 986, Mix7: 1117 sequences).

The amplification specificity of the two new LSU primer sets was tested by *in silico* PCR using the ecoPCR program [56]. Tests were conducted against sequence data for Planta/Fungi (EMBL release, April 2011), Invertebrata, and Bacteria (EMBL release, June 2010). To simulate different levels of stringency, we allowed up to two mismatches between each primer and the template except in the last three bases at the 3′ terminus.

**Figure 1:**
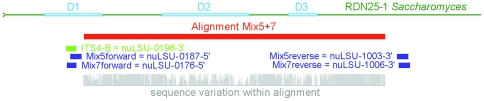
Location of the new Mix5 and Mix7 primer pairs in relation to the ITS4B primer and the 28S (LSU) RDN25-1 gene from the *Saccharomyces cerevisiae* genome, with the more diverse D regions annotated. The ClustalX conservation score for the Mix5+7 alignment is shown in grey.

The location of the new primers relative to the D1, D2, and D3 regions of the LSU gene were determined by comparison to the secondary structure models developed by Hassouna et al. [42], Gutell et al. [57], Srikantha et al. [22], and Gillespie et al. [44]. Primer names consistent with the nomenclature proposed by Gargas & DePriest [58] with a suffix denoting the target fungal group (“Ag” in the case of Agaricomycotina) were created by comparison to the *Saccharomyces cerevisiae* gene RDN25-1 ( = LSU) obtained from www.yeastgenome.org [59]. The relative sequence variation within an alignment containing all amplicons from Mix5 and Mix7 primers was retained from the provided display in ClustalX2 and stems from the mean of sequence distances as defined by that program. The position of the four new 28S primers within the aligned reference gene of *S. cerevisiae* is shown in Figure 1. The new 28S forward primers are located in the 5′ section of the LSU gene and overlap with the ITS reverse primer (ITS4B).

### Testing the new primers on DNA from fungal cultures and soil samples

The PCR efficiency of the newly-developed 28S rDNA primers was tested using a total of 24 fungal pure cultures (19 Basidiomycota, 3 Ascomycota, and 2 Zygomycota; see Table S1), and 2 spore DNA extracts from the phylum Glomeromycota (*Glomus geosporum* and *G. intraradices*) [60]. Soil samples (upper mineral Ah horizon) were collected in April 2008 from three *Fagus sylvatica*-dominated forest sites in the Hainich-Dün Biodiversity Exploratory in western Thuringia, Germany (www.biodiversity-exploratories.de).

Fungal material from agar plate cultures or submerged cultures of zygomycetes was crushed under liquid nitrogen using a pestle and 1.5 ml microcentrifuge tubes. Total genomic DNA was extracted from up to 100 mg of homogenized fungal mycelia using the DNeasy Plant Mini Kit (Qiagen GmbH, Hilden, Germany) according to the manufacturer's protocol. Soil DNA was extracted from 10 g homogenized samples using the MoBio PowerMax Soil DNA Isolation Kit (MoBio Laboratories Inc., Carlsbad, CA, USA) according to the manufacturer's instructions. The DNA extracts were quantified with a NanoDrop ND-1000 UV-Vis Spectrophotometer (NanoDrop Technologies Inc., Wilmington, DE, USA). Where necessary, the template DNA was diluted to a final concentration of 20 ng total DNA µl^−1^. The DNA extracts and dilutions were tested in ITS and LSU amplification and stored at −20°C until further use.

All ITS and LSU amplification reactions were performed using a Mastercycler 5333 (Eppendorf AG, Hamburg, Germany) using GoTaq Green Master Mix (Promega GmbH, Mannheim, Germany) under similar thermocycling conditions (see Table 1). The PCR reactions were performed in a total volume of 50 µl containing 2.5 µl DNA template, 22 µl GoTaq Green Master Mix, and 2.5 µl of a 25 µM equimolar solution of the two primers. All amplified DNA fragments were visualised by 1% agarose gel electrophoresis with ethidium bromide staining.

### Cloning and sequencing

The ITS and LSU amplification products obtained from soil DNA extracts were purified directly with a QIAEX II Gel Extraction Kit (Qiagen). The purified amplicons were ligated into a pCR4-TOPO vector and cloned in TOP10 chemically competent *Escherichia coli* from the TOPO TA Cloning Kit (Invitrogen Life Technologies, Karlsruhe, Germany). Transformed cells were plated out in three dilutions (50 µl, 75 µl and 100 µl per plate) and grown overnight at 37°C on LB agar plates. All plates yielded similar clone numbers. At least 48 white colonies from each sample were subjected to colony PCR using the M13F and M13R primers and a PCR program involving an initial 10 min denaturation step at 94°C, followed by 32 cycles of denaturation (40 sec at 94°C), annealing (30 sec at 54°C), and elongation (40 sec at 72°C), with a final elongation step of 4 min at 72°C. Positive PCR products were treated with ExoSAP-IT (USB Europe GmbH, Staufen, Germany) at 37°C for 15 min to remove undesirable dNTPs, residual primers, and irrelevant single stranded DNA, and were then incubated at 80°C for 15 min to inactivate the ExoSAP-IT. Approximately 45 cleaned amplicons per library were bidirectionally sequenced using an ABI PRISM 3730xl Genetic Analyzer (Applied Biosystems, Darmstadt, Germany) and the Big Dye Terminator v. 3.1 Cycle Sequencing Kit (Applied Biosystems). Sequences were edited using Sequencher v. 4.8 for Windows (Gene Codes Corporation, Ann Arbor, MI, USA) to construct contigs from the manually corrected forward and reverse DNA sequences after removal of primer residues. A total of 40 high-quality sequences per sample and primer pair were selected randomly and pooled by primer set to create three initial 120-clone databases (ITS, Mix5, and Mix7). The number of sequences per dataset was derived from a simulation, an example of which is given in file S2 and Figure S1. DNA sequences are available in EMBL/NCBI GenBank/DDBJ under accession numbers FR750567-FR750682 (ITS) and FR750683-FR750922 (LSU amplified with Mix5 and Mix7).

### Sequence identification and alignment

A perl script (File S1) was used to augment sequence names with relevant information from the NCBI taxonomy path and the best hit from a BLASTn search against the DDBJ sequence database excluding environmental sequences. The “best” hit was selected by examining the top ten BLAST hits for each query sequence, excluding those from non-vouchered specimens, and then selecting that with the highest score and an E value equal or close to zero. BLAST was also used to check whether it would be necessary to exclude and replace non-target sequences in order to obtain 40 sequences per soil sample and 120 per primer pair. Four different alignments were prepared: ITS (120 sequences), Mix5 (120 sequences), Mix7 (120 sequences), and the Mix5+7 overlap (240 sequences). These were created using the program MAFFT v. 6.717 (L-INS-i) [61]. ITS sequences within alignments were checked for putative chimeras using the UNITE PlutoF Chimera checker [62] and the Chimera Test developed in the Fungal Metagenomics Project at the University of Alaska (https://biotech.inbre.alaska.edu/fungal_portal/?program=chimera_test). All LSU sequences were also compared to the LSU database made available by the Alaskan Fungal Metagenomics Project and using MOTHUR v.1.16.0 [63]. The alignments were then manually checked and long overhangs were trimmed. We manipulated the resulting T1 alignments in two ways. In the first treatment, we replaced all remaining leading and trailing indel symbols (−) with the ambiguity code N to denote the presence of a gap or an unspecified nucleotide (yielding the T2 alignments). In the second treatment, we replaced leading and trailing indel symbols with N and also excluded ambiguously alignable areas from further analysis (generating the T3 alignments). The 12 resulting alignments, termed Mix5 T1, 2, 3, Mix7 T1, 2, 3, Mix5+7 T1, 2, 3, and ITS T1, 2, 3 (Table 2) are available in NEXUS format from the Dryad Digital Repository (doi:10.5061/dryad.m95s7sq1).

**Table 2:** Characteristics of alignments.

Alignment	AverageA, C, G, T frequencies (%) after BioEdit	Average sum of ambiguous base frequency (%)	Gaps (%) after BioEdit	Alignment length (bp)	Positions with H>0 and H = 0 left*	k_1_, k_2_, R values**after MEGA	Diversity parameters for 0.05 OTU cutoffcalculated using FastGroupII		
							OTU number	Chao1*** (ribotypes)	Shannon**** (nats)
ITS T1	19.15, 15.87, 17.57, 21.96	0.01(K+M+S+Y)	25.43	1073	242, 194	2.32, 9.52, 2.62	35	323.00	2.86
ITS T2	19.15, 15.87, 17.57, 21.96	0.31(K+M+N+S+Y)	25.13	1073	345, 194	2.32, 9.52, 2.62	35	323.00	2.86
ITS T3	30.65, 19.36, 22.71, 24.65	0.81(K+M+N+S+Y)	1.82	405	203, 184	2.59, 3.51, 1.21	22	52.25	2.48
Mix5 T1	24.34, 19.58, 28.50, 22.20	0.01(R+W)	5.36	809	278, 401	0.54, 11.96, 2.42	20	36.00	2.47
Mix5 T2	24.34, 19.58, 28.50, 22.20	0.07(N+R+W)	5.30	809	320, 401	0.54, 11.96, 2.42	24	40.67	2.63
Mix5 T3	25.68, 19.09, 29.45, 23.30	0.07(N+R+W)	2.41	687	272, 388	2.54, 12.73, 3.07	16	25.00	2.17
Mix7 T1	24.95, 19.70, 29.28, 22.22	0.03(K+N+R+S+W)	3.82	819	275, 437	24.68, 27.88, 12.69	21	57.00	2.06
Mix7 T2	24.94, 19.70, 29.30, 22.22	0.25(K+N+R+S+W)	3.59	819	314, 437	24.68, 27.88, 12.69	19	44.00	2.00
Mix7 T3	25.78, 19.07, 30.07, 23.44	0.29(K+N+R+S+W)	1.35	700	267, 419	23.49, 34.78, 13.51	14	20.25	1.81
Mix5+7 T1	24.39, 19.58, 28.49, 22.10	0.01(R+S+W)	5.43	811	328, 346	12.24, 17.81, 6.88	33	81.17	2.49
Mix5+7 T2	24.39, 19.58, 28.49, 22.10	0.06(N+R+S+W)	5.38	811	370, 346	12.24, 17.81, 6.88	33	58.60	2.48
Mix5+7 T3	25.51, 19.09, 29.41, 23.37	0.07(N+R+S+W)	2.55	687	322, 336	0.33, 20.80, 3.90	21	41.25	2.10

*The two numbers in this column denote the number of positions in each alignment that retain phylogenetic information (entropy > 0) and that have none (entropy = 0) after deleting gap-containing sites;

**k_1_ values give the transition∶transversion rate ratio for purines; k_2_ values do the same for pyrimidines. The overall transition/transversion bias is R  =  [A*G*k_1_ + T*C*k_2_]/[(A+G)*(T+C)];

***statistical richness estimator [67],

****Shannon-Wiener diversity index [66].

### Statistical and phylogenetic analyses

The online tool FastGroupII [64] was used for dereplicating and rarefying the sequences within the 12 alignments described above. This was achieved using the Percentage Sequence Identity algorithm [65] with OTU cutoff levels of 95%, 97%, 98%, and 99% (or equivalently, 0.05, 0.03, 0.02, and 0.01, respectively), ignoring gap-containing alignment columns. The evenness, the Shannon-Wiener index [66], and the Chao1 index [67] for each alignment were calculated, using FastGroupII in the latter two cases. Neighbour-joining (NJ) was performed with the BioNJ algorithm [68] as implemented in SeaView v. 4.2.3 [69], using the Kimura 2-parameter model (K2P) [70] and 100 bootstrap pseudoreplicates. As before, gap-containing columns were excluded. Dendroscope v. 2.6.1 [71] and Serif PhotoPlus SE (Serif (Europe) Ltd, Nottingham, UK) were used to generate circular representations of the phylogenetic compositions of the ITS T1, Mix5 T1, and Mix7 T1 alignments (using a 95% OTU cutoff in each case). Average bootstrap support values were calculated for supported higher taxonomic groups. For the OTUs identified using a FastGroupII cutoff of 95%, a test was conducted to see whether the names assigned on the basis of BLAST searches coincided with monophyletic groups. Thus, minimum-evolution (ME) phylogenies were calculated in MEGA v. 4.0.2 [72], using 100 bootstrap pseudoreplicates, deletion of gaps, and the K2P base substitution model (uniform rates among sites). Further phylogenetic and molecular evolutionary analyses were conducted using MEGA. Graphical representations of data were prepared using Corel Photo Paint X3 and Corel Draw X3 (Corel Corporation, Ottawa, ON). All alignments were tested for substitution saturation [73] by plotting the number of transitions and transversions against the K2P distance using the DAMBE v. 5.2.9 software package [74].

Information entropy [75] can be seen as an indicator of the informativeness of an alignment column. We investigated the entropy landscape of each alignment using the modified Shannon entropy formula described below. The overall entropy (in units of nats, which is equivalent to nits or nepits) at position i (H_i_) was calculated as the negative sum of the frequencies (i.e. probabilities, p) of residues occurring at that position, multiplied by their natural logarithm: 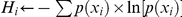, where p(x_i_) is the frequency of residue x (which may be A, C, T, G or a real indel, E) at position i in the alignment. We used different feeder formulae to independently determine the frequencies of each residue at a specific position in our alignments, using the IUPAC ambiguity codes (e.g. 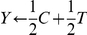, 
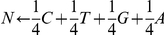
, etc). A gap in an alignment could be due to missing information (and could thus be filled by any one of the four standard nucleotides in reality) or could indicate a real indel position. Arbitrarily, all of these 5 possibilities were assigned equal frequencies. For example, the partial entropy of cytosine (C) at position i was calculated as follows: 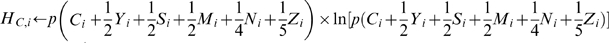. The expression used for real gaps was: 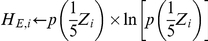, where Z_i_ is the gap in the alignment at position i and could actually be A, C, T, G or a real indel. Because ln(0) is undefined, partial entropies for non-occurring character states were not added, which also means that different alignment positions have different maximum possible entropy values. However, the product of a value that is close to zero and its own natural logarithm is extremely small, so this did not greatly affect the calculated entropy.

## Results

### Specificity of the new 28S rDNA primers

Virtual PCR tests of new LSU primers using ecoPCR and the GenBank-derived databases confirmed their specificity for Agaricomycotina and demonstrated their robust exclusion of non-fungal rDNA. In tests against the Planta/Fungi database, which features 2 014 974 non-environmental nucleotide sequences, Mix7 always amplified Agaricomycotina sequences exclusively even under very relaxed *in silico* PCR conditions (two mismatches between primer and target sequence allowed). Mix5 exhibited similar specificity if no mismatches were allowed; if two mismatches were allowed, two Ascomycota and three Ustilaginomycotina sequences were amplified, representing 0.4% of all virtual amplicons. When tested against Invertebrata and Bacteria databases, neither primer set yielded any amplification even when allowing two mismatches.

Of the 26 pure fungal DNA templates tested *in vitro*, all 19 Basidiomycota (represented by 8 Agaricales, 2 Boletales, 7 Polyporales, and 2 Russulales species) were amplified with both primer pairs (Table S1). While Polyporales amplification had not been intended, it was not deemed problematic because polypores might occur on litter fragments such as sticks, and litter dwellers were the target ecological group when designing the primers. Amplicons of ca. 800 bp were obtained independently for various concentrations of template DNA (undiluted and for dilutions containing 5 to 50 ng of total DNA µl^−1^), which was considered promising in terms of the potential for robust amplification from environmental extractions. Ascomycota, Glomeromycota, and Zygomycota were not amplified.

### Soil sample DNA amplification efficiency and 28S rDNA sequence variability

We tested each primer pair for its ability to amplify fungal targets from environmental samples and for evidence of non-target amplification (Table 1). All tested rDNA primers preferentially amplified DNA from the target fungal taxa (100% of all basidiomycete ITS sequences and 97–98% of all Agaricomycotina LSU sequences) while effectively excluding plant and non-target fungal sequences in soil samples. Undesired plant sequences (94% identity to Euphorbiaceae 26S rRNA reference genes from GenBank) were only detected using the Mix7 pair after expanding the cloning approach in subsequent studies (3 of 1117 high quality sequences, data not shown). Fungal misamplifications (non-Agaricomycotina LSU sequences) occurred on a relatively small scale and only for Mix5 (0.2% non-Agaricomycotina sequences). A single atypical sequence was identified among the 120 amplified using basidiomycete-specific ITS primers; the anomalous sequence originated from a mitosporic Agaricomycotina genus (*Rhizoctonia*). No non-target sequences that would have to be replaced were detected. 11.7% of the sequences amplified using the Mix5 primers originated from Agaricomycotina orders other than Agaricales, Boletales or Russulales (namely Atheliales, Sebacinales, and Tremellales). 6.7% of the Mix7 sequences originated from Sebacinales and Thelephorales.

Screening for chimeric sequences with different programs revealed relatively little (ITS) or no (LSU) evidence for chimeras. Using the UNITE PlutoF module [76], 17 potentially chimeric sequences were identified among the 120 sequences in the ITS dataset. Detailed examination of these sequences (using discontiguous megaBLAST searches against the NCBI GenBank nucleotide database) led us to reject 13 as false positives; the remaining 4 were accepted to be putative chimeric sequences (*Russula/Inocybe*, *Inocybe/Russula*, *Lactarius/Xerocomus*, and *Xerocomus/Trechispora*). These were removed from the original ITS alignments and excluded from subsequent phylogenetic analyses. The Chimera Test of the University of Alaska did not identify any chimeric sequences in the ITS dataset. Similarly, no potential chimeras were identified in the LSU data using the chimera checker at the Alaskan website or the MOTHUR Chimera Slayer tool.

The fungal 28S rDNA fragments amplified *in vitro* from soil samples by both newly developed LSU primer pairs (i.e. the Mix5+7 alignment of 240 sequences) included the last third of the D1 expansion domain and the entirety of the D2 and D3 domains. The greatest variation in the amplified LSU sequences was found at and near these divergent regions, which accounted for over 45.3% of the total length of the Mix5+7 alignment. The ClustalX quality scores (shown in grey in Figure 1) for this alignment indicated that most of the sequence variability for this alignment (i.e. the lowest conservation scores) occurred at the 3′ end, and around the D1, D2, and D3 regions.

### Alignment modification and the influence of the OTU cutoff on rarefaction analysis and OTU characteristics

Compared to the LSU datasets, the ITS alignments contained more gaps (which accounted for >25% of all sites in the T1 and T2 alignments) and ambiguous character states (more than 0.8% of all sites in the T3 alignment). As expected, deleting gaps and marginal ambiguous sites from the original ITS alignment (which together accounted for more than 66% of the alignment's length) resulted in a major loss of phylogenetic information (77% removal of informative sites; see Table 2).

Deleting ambiguous sites (T3) had a strong impact in terms of decreasing diversity for all primer pairs (at a cutoff level of 95%, i.e. 0.05; see Table 2). Interestingly, within individual ITS datasets, replacing the leading and trailing ends with N did not change the values of the diversity indices. Conversely, for the LSU datasets, these parameters took different values under each treatment (with the exception of the OTU number for Mix5+7, which was the same in the T1 and T2 alignments). Generally, the Chao1 index and the OTU number seemed to be more dependent on the alignment treatment than the Shannon-Wiener index, which did not change by more than 0.3 units within a single dataset, going from 2.47 for Mix5-T1 to 2.17 for Mix5-T3. This effect was especially pronounced for the ITS dataset: the Chao1 index decreased more than six-fold on going from T1/T2 to T3 (while the Shannon-Wiener index decreased by less than 13%). The ITS dataset had 22 OTUs, more than the Mix5 and Mix7 co-alignment. Of the LSU datasets, Mix7 had the lowest Shannon-Wiener index (which took a value of less than 2 for the Mix7-T3 alignment) and exhibited greater treatment-dependent variation than Mix5. Interestingly, at the 95% sequence similarity level, the Mix5 dataset did not follow the pattern shared by all other datasets; the greatest diversity occurred in the T2 alignment rather than its untreated T1 counterpart.

**Figure 2:**
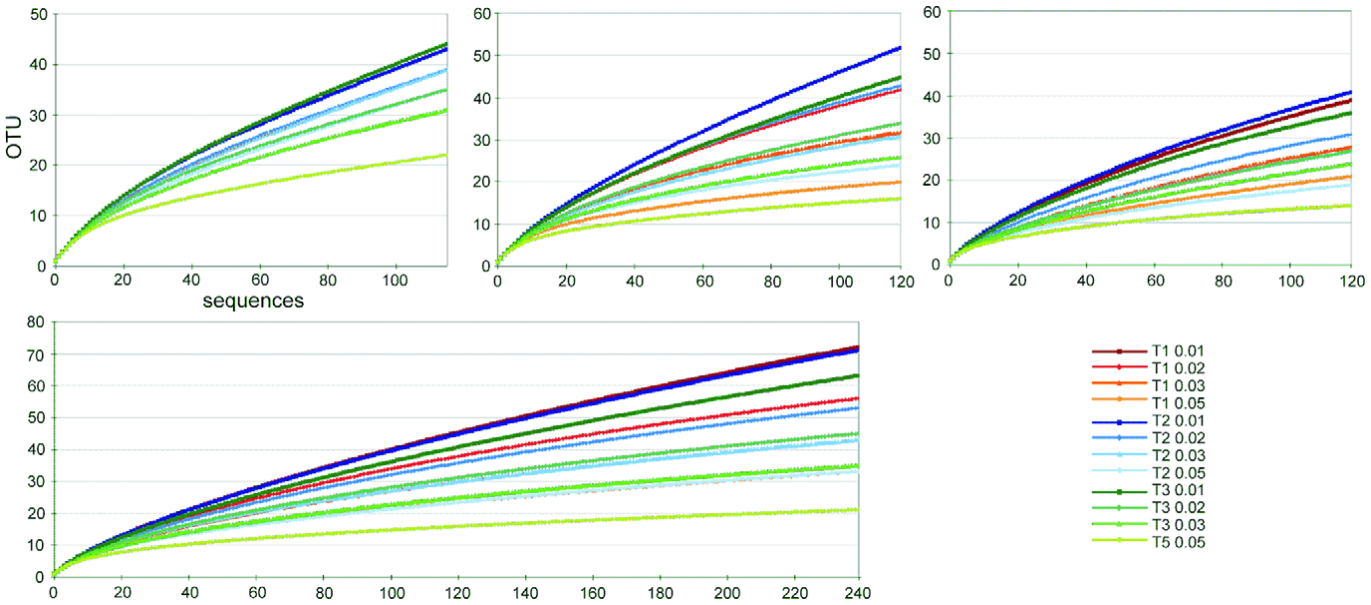
FastGroupII derived rarefaction analysis of 4 sequence datasets using different alignment treatments and cutoff levels: A) ITS, B) Mix5, C) Mix7, and D) a combined Mix5+7 LSU dataset.

The alignment treatment and the OTU cutoff both generally had large effects on FastGroupII rarefaction trends and OTU numbers (Figure 2). The replacement of trailing ends by N in the T2 alignments caused bidirectional cutoff-dependent changes even within the same dataset (e.g. Mix7). In some cases, this increased the number of OTUs relative to the unaltered T1 alignments (this was observed for Mix7-T2-0.01 and Mix7-T1-0.01); in others, the number of OTUs decreased (e.g. for Mix7-T2-0.05 and Mix7-T1-0.05). Deleting ambiguous sites to create the T3 alignments strongly affected the rarefaction curves, intensifying the OTU-cutoff effect and reducing the OTU number. For example, in the case of Mix7 the OTU number decreased from 41 OTUs in T2-0.01 to only 14 OTUs in the T3-0.05 alignment. The T1 and T2 rarefaction curves of the ITS data tracked each other tightly. Conversely, the curves for all LSU and ITS T3 alignments were more widely spaced, clearly illustrating their dependence on the OTU cutoff. Generally the FastGroupII rarefaction curves for the unaltered alignments (T1) were more similar than those for the treated ones. The T3-0.05 alignments consistently had the lowest numbers of OTUs. With the same number of sequences (120) and identical alignment conditions (T2-0.01), the Mix5 primer pair yielded a much higher maximal diversity (52 OTUs) than Mix7 (41 OTUs) suggesting that greater weighting of Mix5 was more influential than Mix7 in the assignment of OTUs within the combined LSU dataset (Mix5+7).

**Figure 3:**
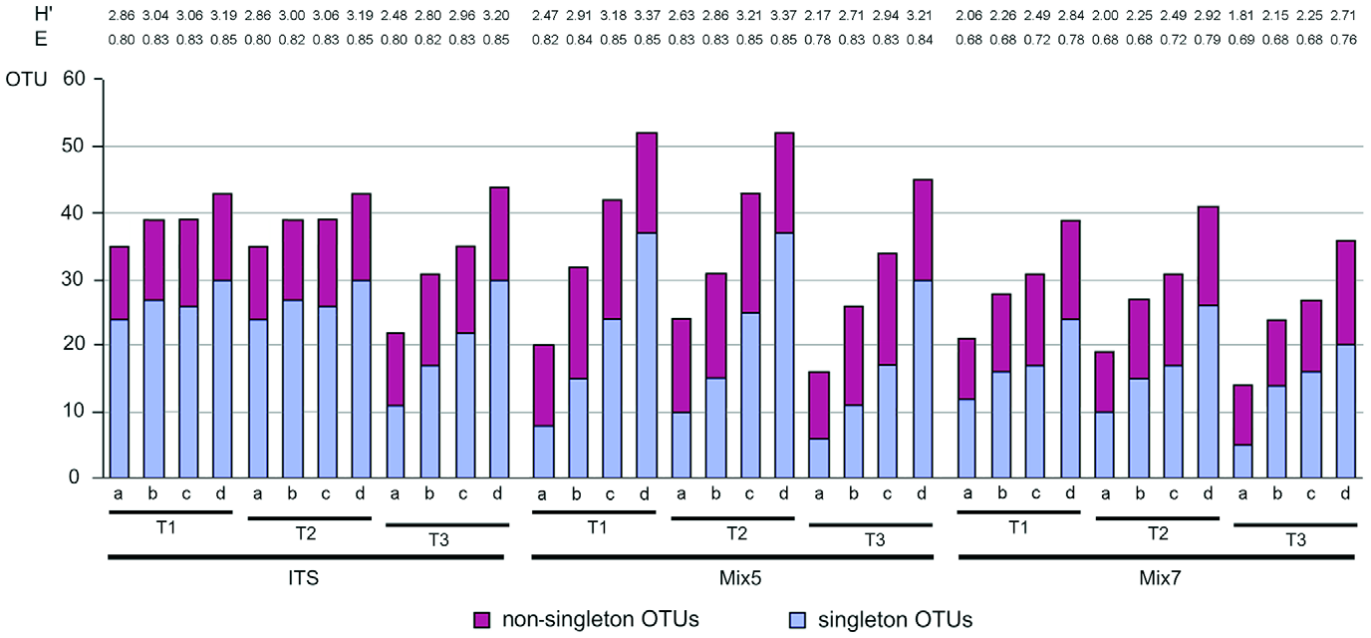
The influence of varying the FastGroup II OTU cutoff and alignment processing on the number of singleton and non-singleton OTUs, the Shannon index (H′) as calculated using FastGroupII, and the species evenness, E = H′/lnS where S is the species richness, i.e. the OTU number. T1 alignments are unprocessed, T2 alignments were generated by replacing the leading and trailing ends with N, and T3 alignments were generated in the same way as T2 but with the removal of ambiguous columns. Cutoff levels were as follows: a = 0.05, b = 0.03, c = 0.02, and d = 0.01.

A mathematical singleton is a unit that contains only one element, e.g. a nucleotide occurring only once in an alignment column. In molecular ecology, the term singleton refers to an OTU that occurs only once in a given dataset, i.e. a singleton OTU. By definition, it thus contains only one individual sequence. Doubletons and higher multitons can also contain sequences that are fully identical. In order to understand a concept introduced later on in this paper, it is important to differentiate between this subtype of multitons (henceforth referred to as quasi-singletons) and conventional multitons. For all alignments and treatments, most of the OTUs were real singletons containing only one sequence (Figure 3). Over all datasets considered, the highest percentages of singleton OTUs were observed for the T1 and T2 treatments at the 0.01 OTU-cutoff level. At all cutoff levels, singleton OTUs were more abundant for the ITS alignments than for the LSU alignments, and the ITS alignments generally contained more OTUs overall than either Mix5 or Mix7. Non-singleton OTUs accounted for 30–50% of the OTUs in the ITS dataset, 29–63% of the OTUs in the Mix5 dataset, and 37–64% of all OTUs in the Mix7 dataset, depending on alignment treatment and OTU-cutoff.

Varying the cutoff level had little effect on the number of OTUs for the ITS alignment but had a much larger effect on the LSU datasets. For both the ITS and LSU datasets, the level of sequence diversity decreased on going from the untreated alignments to the T3 alignments, but this trend was much more pronounced for the LSU data. Within the ITS dataset, the T1 and T2 treatments yielded identical cutoff-dependent OTU numbers. For the LSU data, and especially for Mix5, the ratio of singleton OTUs to total OTUs increased rapidly as the cutoff level was reduced (i.e. as the level of sequence similarity required to group multiple sequences into an OTU increased). Thus, the ratio for T1-0.05 was 0.4 (i.e. 8 of 20 OTUs were singletons) while that for T1-0.01 was 0.7 (37 of 52 OTUs). Conversely, for the LSU data, there was no apparent relationship between alignment treatment and OTU number. Out of all 36 different alignments considered, the Mix5 dataset exhibited the greatest variability in OTU numbers and the highest diversity at the 0.01 OTU-cutoff level. Overall, going from T1 to T2 alignments did not change the OTU number for the ITS datasets but had varied effects depending on the cutoff level with the LSU datasets. Conversely, going from T2 to T3 alignments significantly reduced OTU diversity for all datasets.

The OTU cutoff affected the universal biodiversity indices for the LSU and ITS datasets in similar ways (Figure 3). As the OTU-cutoff decreased, the Shannon-Wiener index (H′) and OTU evenness (E) increased. The maximum diversity values for all primer sets were achieved with an OTU cutoff of 0.01. However, the precise alignment treatment that yielded the highest diversity depended on the primer pair used. For Mix7, it was the T2 treatment (H′ = 2.9, E = 0.8); for ITS, the T3 treatment (H′ = 3.2, E = 0.9); and for Mix5, the T1 and T2 treatments (for both, H′ = 3.4, E = 0.9). This suggests that the treatment has a much weaker effect on diversity than the OTU cutoff. Across all datasets and treatments considered, the Shannon-Wiener index ranged from 1.8 (the lowest value observed with Mix7) to 3.4 (the highest value observed with Mix5) indicating that both the ITS and the LSU datasets exhibited a high level of species evenness and richness, especially in the case of Mix5 (for which the corresponding range was 2.2 to 3.4). For the less diverse ITS and Mix7 datasets, the cutoff-associated OTU evenness (E) seemed to be robust to changes in the treatment of the alignment; for a given cutoff level, the E value remained constant no matter how the alignment was processed (e.g. for the ITS dataset with an OTU cutoff of 0.05, the E value for all treatments was 0.8).

### Comparing the Agaricomycotina diversity detected using ITS and LSU primers

Different primer sets yielded similar but not identical levels of diversity. The ITS primers showed the greatest affinity for the primary target orders – the Agaricales, Russulales, and Boletales DNA (96.7% of all sequences). These three Agaricomycotina orders were heavily represented in all datasets, but LSU primers also efficiently amplified sequences from the Sebacinales (especially Mix5, for which this order accounted for 9.2% of all identified sequences and 10% of all OTUs), which were not represented in the ITS data.

**Figure 4:**
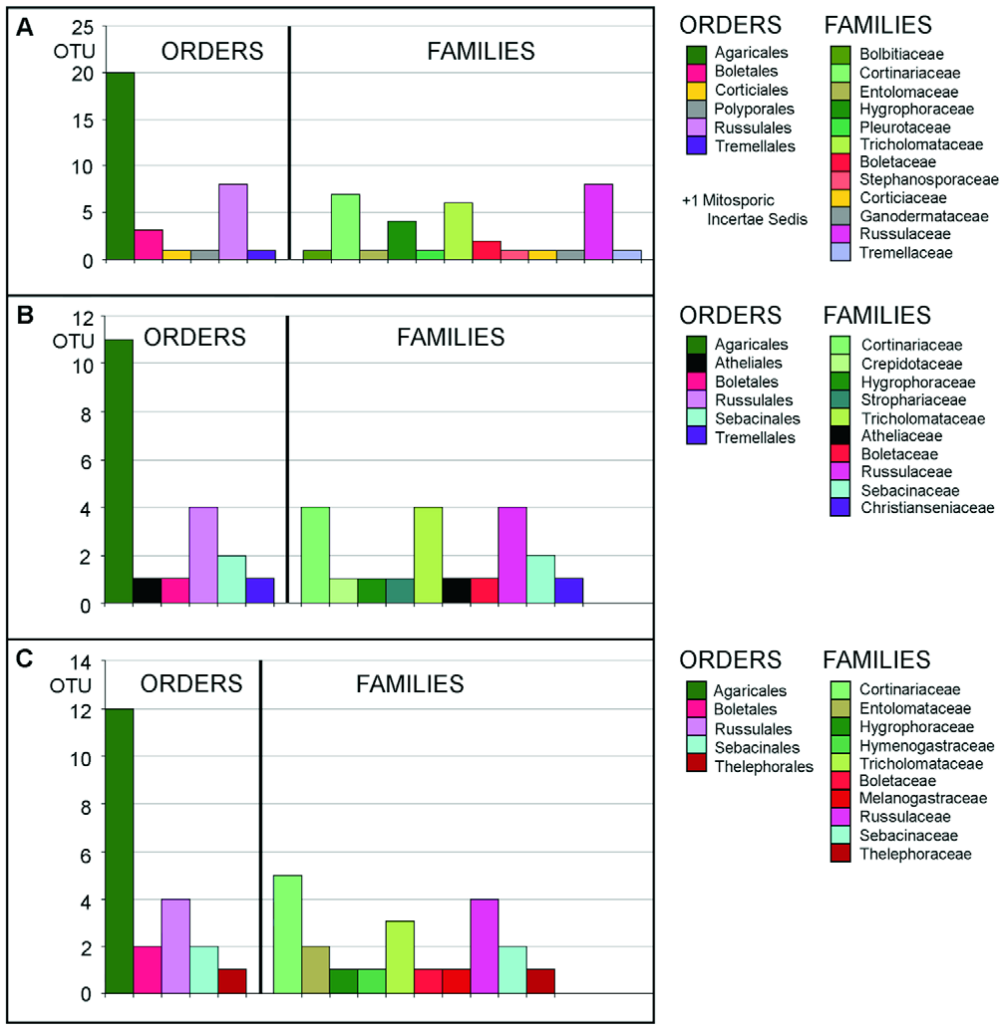
High-level (family and order) diversity of the FastGroupII derived OTUs for the T1-0.05 alignments (5% OTU cutoff, gaps and ambiguities not treated) for A) ITS, B) Mix5, and C) Mix7.

The Agaricales overwhelmingly dominated all datasets, as indicated in Figure 4 (they accounted for 57.0% of all ITS OTUs, 55.0% of Mix5 OTUs, and 57.1% of all Mix7 OTUs identified at the 95% sequence similarity level). Within the Agaricales, three families (Cortinariaceae, Hygrophoraceae, and Tricholomataceae) were predominant in all datasets (accounting for 50.0% of all ITS, 45.0% of all Mix5, and 42.9% of all Mix7 OTUs). Conversely, the Bolbitiaceae and Pleurotaceae were detected only with ITS primers, the Crepidotaceae only with Mix5, and the Hymenogastraceae only with Mix7. The overall number of orders (5 with Mix7, 6 with ITS and Mix5) and families (10 for each of the LSU datasets and 12 for the ITS dataset) detected was similar for all datasets, save that the ITS dataset contained a greater number of families with only a single OTU (6 OTUs were only detected with the ITS primers, compared to 3 for each of the LSU primer sets). These differences were reflected in the higher taxa identified in each dataset. Only the ITS primers amplified sequences originating from the Corticiales and Polyporales. Similarly, only Mix5 amplified Atheliales sequences and only Mix7 amplified those from Thelephorales. All families and orders detected by only one primer set were represented by only a single OTU. Excluding these taxa would drastically reduce the overall diversity of all analysed datasets and make the OTU distributions for the different primer sets very similar.

### Correlations between BLAST derived taxonomy (OTU names) and bootstrap-supported monophyletic clades

**Figure 5:**
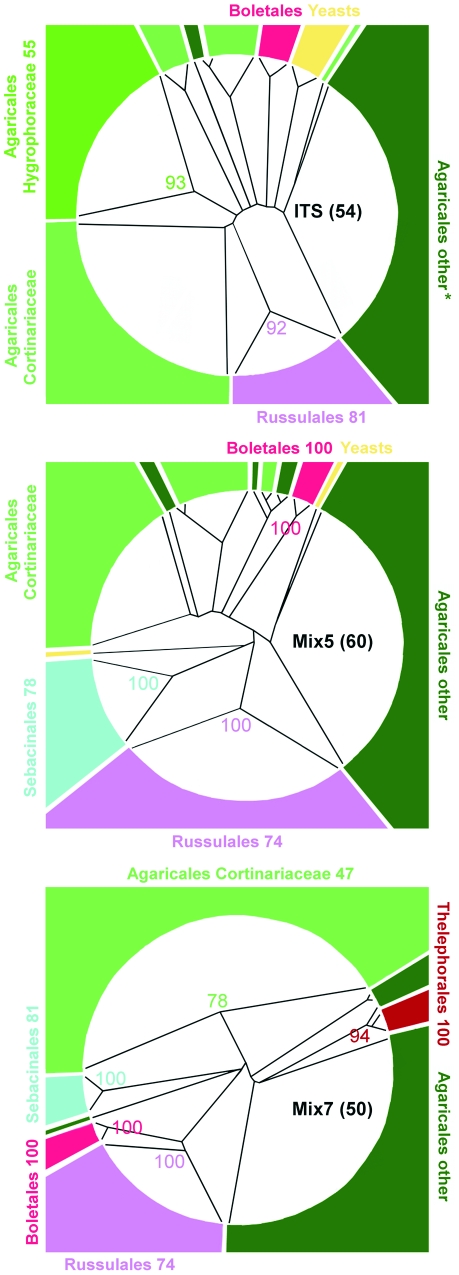
Recovery of higher-level phylogenetic groups with OTUs identified using BLAST searches for the T1-0.05 alignments. Neighbor-joining was performed with the BioNJ algorithm as implemented in SeaView v.4.2.3 using the K2P model and 100 bootstrap pseudoreplicates; gaps were ignored. Dendroscope v.2.6.1 was used to prepare circular representations of the phylogenetic trees. Bootstrap values for supported groups are given at the appropriate node, along with average bootstrap values for supported higher taxonomic groups (internal average bootstrap scores are shown under the appropriate taxon label) and across the entire alignment (in the center of the tree). The width of each coloured block close to the circumference of the trees is exactly equal to the number of individual sequences representing the appropriate phylogenetic group; * OTU contains an ITS sequence of uncertain taxonomy (MycoBank MB19330: Agaricales, NCBI Taxonomy ID 178442: Boletales, Index Fungorum LSID 19330: Russulales) that makes the monophyly of the Boletales clade questionable (and therefore not bootstrap supported in the ITS tree).

The fungal diversity revealed by the three primer sets was compared at higher taxon levels using circular BioNJ-K2P trees generated using SeaView (Figure 5) with three calculated bootstrap parameters: the average bootstrap score for the entire tree (i.e. the arithmetic mean of all the tree's bootstrap values), the number of bootstrap scores above 49 for each supported clade, and the average internal node bootstrap support for higher taxa that appear to be monophyletic (the arithmetic mean bootstrap value across all internal nodes for each supported clade). Remarkably, none of the analyzed alignments yielded the expected phylogenies. The main problem was that the Boletales (ITS and Mix5) or Thelephorales (Mix7) were located between the agaric sequences, making the Agaricales non-monophyletic in every alignment. For ITS, the only order recovered monophyletically and with a high degree of confidence was the Russulales (92% support, compared to an average of 81%). This clade was monophyletic in all three diagrams (ITS, Mix5, and Mix7), but was better supported in the LSU trees (100%, average 74%). Notably, an ITS sequence of uncertain taxonomy [77] derived from the genus *Stephanosphora* (which was previously detected by Porter et al. [40] in an environmental sequencing study targeting the LSU) made the monophyly of the Boletales in the ITS tree look questionable, despite this being the most robustly supported clade in both LSU alignments (Figure 5). Of the two sets of LSU primers examined, the Mix7 pair yielded monophyletic assemblages for the greatest number of orders (Boletales, Russulales, Sebacinales, and Thelephorales). Interestingly, all Mix7 sequences representing the Cortinariaceae clustered monophyletically. Conversely, in the Mix5- and ITS-derived trees, the Cortinariaceae were intersected by other agaric sequences. Average levels of bootstrap support over the entire tree were generally low for all datasets (50 for Mix7, 54 for ITS, and 60 for Mix5), but some well-supported clades were detected in both LSU datasets, notably the Boletales (100%, average 100%), Russulales (100%, average 74%), and Sebacinales (100%, average 78–81%).

**Figure 6:**
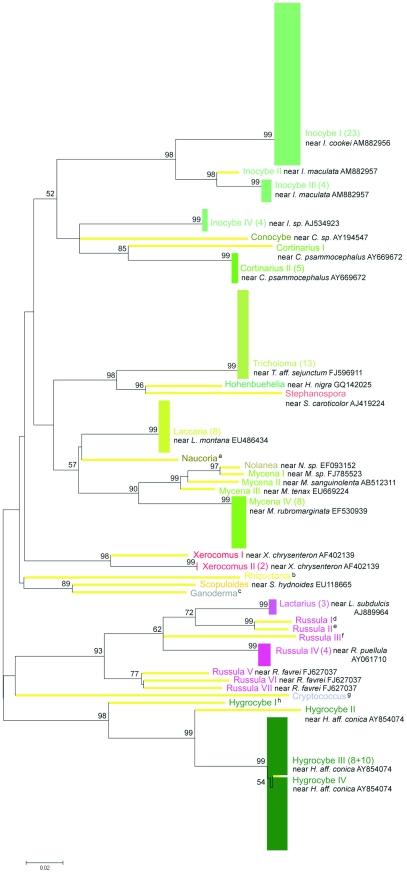
MEGA 4.0.2-derived phylogenetic relationships between sequences within the ITS T1-0.05 dataset at the FastGroupII-defined OTU level (unrooted ME trees, 100 bootstrap replicates, K2P model). Bootstrap values >50 are highlighted. OTU names include the genus, number of sequences, and most relevant BLAST hit(s) with NCBI annotation(s): a = near *Naucoria escharoides* AY900084, b = near *Rhizoctonia sp.* Eab-S1 AJ242881, c = near *Ganoderma lipsiense* EF060006, d = near *Russula cyanoxantha* AY606960, e = near *R. sp.* MHM078 EU569265, f = near *R. farinipes* DQ421983, g = near *Cryptococcus podzolicus* AJ581036, h = near *Hygrocybe aff. conica* PBM 918 AY854074. Singleton OTUs are denoted by thick pale branch lines; multisequence OTUs are represented as blocks (their widths correspond to their maximum internal distance).

**Figure 7:**
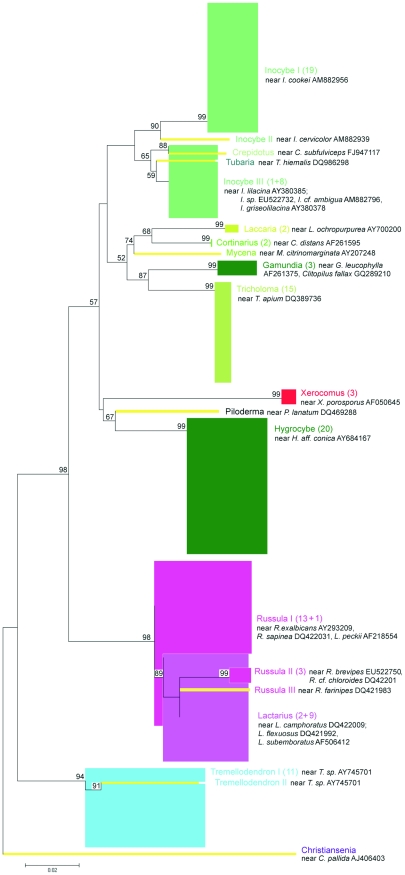
MEGA 4.0.2-derived phylogenetic relationships between sequences within the Mix5 T1-0.05 dataset at the FastGroupII-defined OTU level (unrooted ME trees, 100 bootstrap replicates, K2P model). Bootstrap values >50 are highlighted. OTUs are named and represented as in Figure 6.

**Figure 8:**
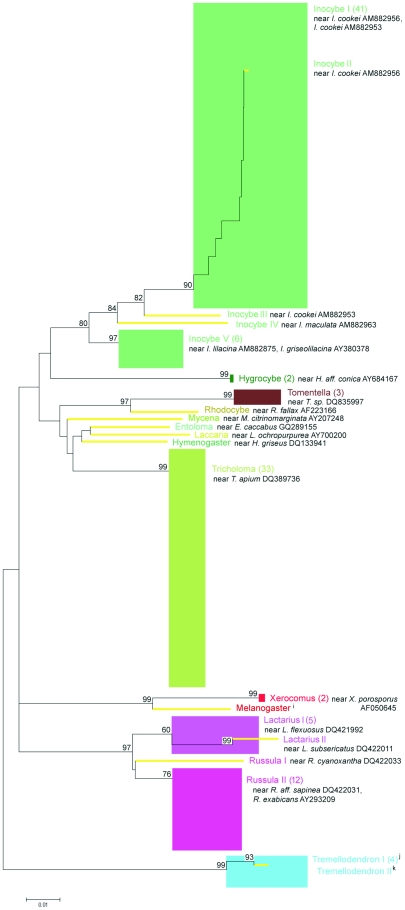
MEGA 4.0.2-derived phylogenetic relationships between sequences within the Mix7 T1-0.05 dataset demonstrated at the FastGroupII-defined OTU level (unrooted ME trees, 100 bootstrap replicates, K2P model). Bootstrap values >50 are highlighted. OTUs are named and represented as in Figure 6; i = near *Melanogaster variegatus* DQ534668, j, k = near *Tremellodendron sp. PBM2324* AY745701.

More detailed fungal diversity analyses performed at the OTU level (ME-K2P phylogenies) demonstrated that the BLAST derived OTU names corresponded to MEGA-derived monophyletic groups (Figures 6, 7, 8). With a 95% sequence similarity cutoff, 35 OTUs (ca. 68.6% of which were singletons) were identified in the ITS clone library, compared to only 20 (40% singletons) for Mix5 and 21 (57.1% singletons) for Mix7. All datasets were overwhelmingly dominated by sequences identified by BLAST as deriving from the genus *Inocybe* (27.6% of ITS, 24.2% of Mix5, and 41.7% of Mix7 sequences). The most abundant OTU across all datasets, *Inocybe cookei* (BLAST-derived accession number AM882956 contained both the ITS and the LSU sequences), accounted for 19.8%, 15.8%, and 44.4% of the sequences in ITS, Mix5, and Mix7 data, respectively. Phylogenetic resolution was problematic for *Russula*-like sequences, indicating that multiple BLAST-derived names may have been assigned to one FastGroupII-derived LSU OTU or that many poorly-supported ITS OTUs represented the same BLAST hit. Only *Laccaria* and strongly represented *Tricholoma* sequences were all grouped into one maximum bootstrap supported OTU each but these were not conspecific across all three datasets. These two groups (which were identified as *L. montana* and *T. sejunctum* using the ITS data but as *L. ochropurpurea* and *T. apium* using the LSU data) were conspecific for both LSU alignments. Some of the non-singleton OTUs identified from the Mix5 sequences using FastGroupII fell within other OTUs. In particular, *Lactarius* and *Russula* sequences were mixed even within the same OTU. For example, the *Russula* I OTU featured 13 *Russula* sequences and 1 *Lactarius* sequence. The obvious difference between the ITS- and LSU-derived ME-K2P trees was the large internal distance of the LSU OTUs. ME bootstrap analysis of the ITS data generated only 54.8% interior nodes with over 49% bootstrap support (some of these values for the FastGroupII-delimited OTU level are shown in Figure 6) and only 17.9% of all nodes achieved maximum bootstrap values of 99%. Conversely, for the LSU data, 76.1% of Mix5 nodes and 67.6% of Mix7 nodes had over 49% bootstrap support (maximum values of 99% bootstrap support in 19.3% and 21.1% of nodes , respectively).

### Substitution saturation and entropy

**Figure 9:**
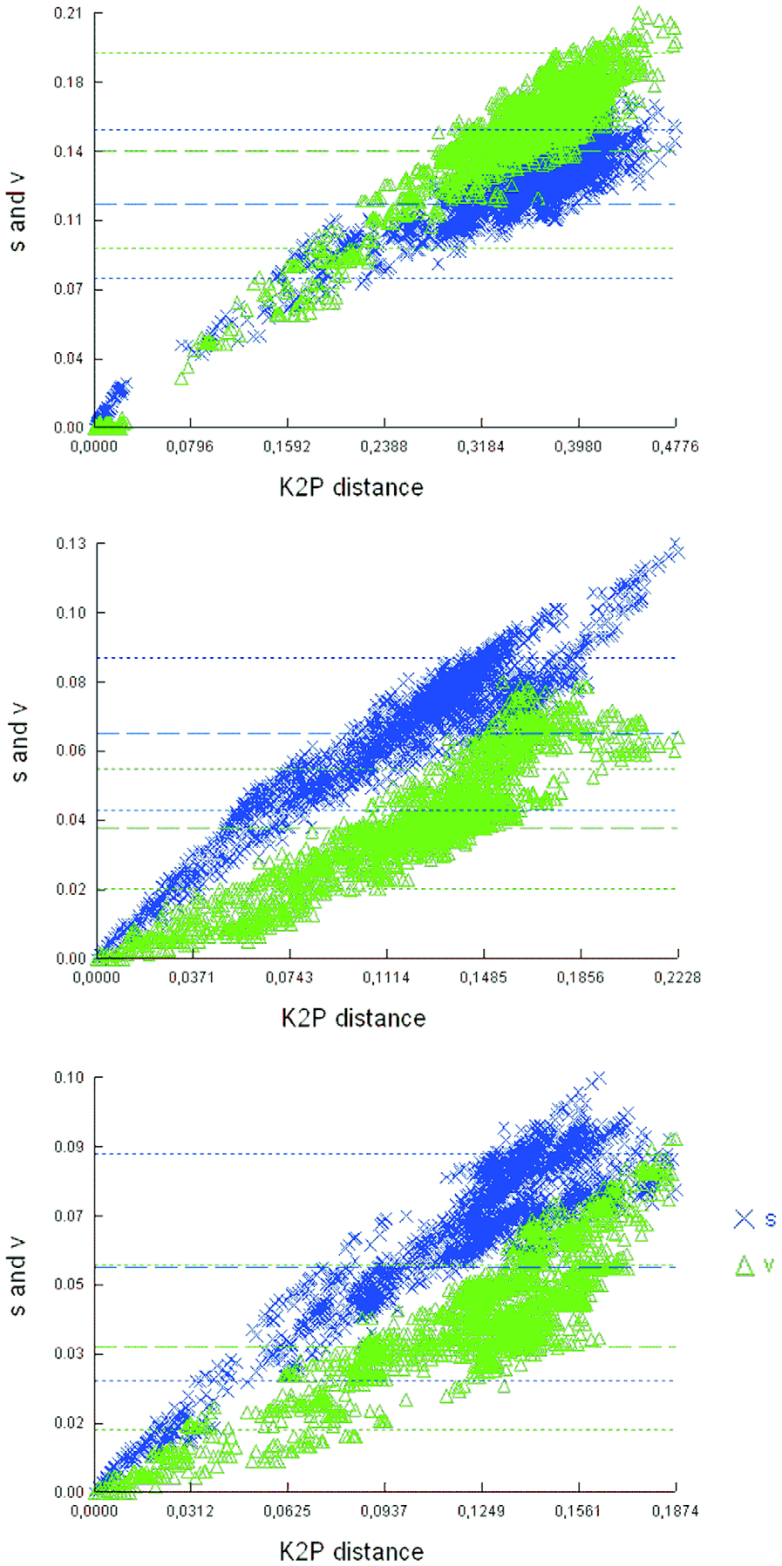
DAMBE substitution saturation plots for A) ITS, B) Mix5, C) Mix7 sequence datasets (unaltered T1-0.05 alignments). The number of transitions (s) and transversions (t) is plotted against the K2P distance; lines indicate the mean values (thick lines) and standard deviations (fine lines) of s and v.

Figure 9 illustrates how the number of observed transitional and transversional substitutions gradually increased with divergence for all ITS and LSU datasets. The number of mutations (transitions and transversions) was highest in the ITS dataset, with transitions being more common than transversions. Substitution saturation occurs when the frequency of transitions overtakes the frequency of transversions. Unlike Mix5 and Mix7, the ITS alignment reached mutation saturation at a K2P distance of approximately 0.2.

We attempted to find a simple parameter that would describe the informativeness of an alignment and could be used to compare whole alignments or subsections thereof to one-another. Starting with the cumulative entropy H (the stacked entropy across all alignment columns), we defined the *relative heterogeneity* (h) as the ratio of the actual entropy (H_i_) to the cumulative maximum (potential) entropy, H_max_; h = H_i_/H_max_. A relative heterogeneity of 0 means that the alignment is very homogenous and that all columns contain only one character state each. This is the easiest alignment to make, but it carries no entropy (i.e. it contains no information and thus no phylogenetic information) on its own. A relative heterogeneity of 1 does not indicate that an alignment is chaotic but that all character states are equally distributed. Therefore, assuming that there is no differential weighting of the various substitution types, no alignment column provides any resolving phylogenetic information that can be used in distance- or likelihood-based methods. The relative heterogeneity correlates with average and median entropy values, which also tend to be low for alignments with little phylogenetic information. However, alignments with relative heterogeneities between 0 and 1 contain information that may be phylogenetically relevant. Low heterogeneity values are typically indicative of large numbers of uni-character alignment columns with no phylogenetic information.

**Figure 10:**
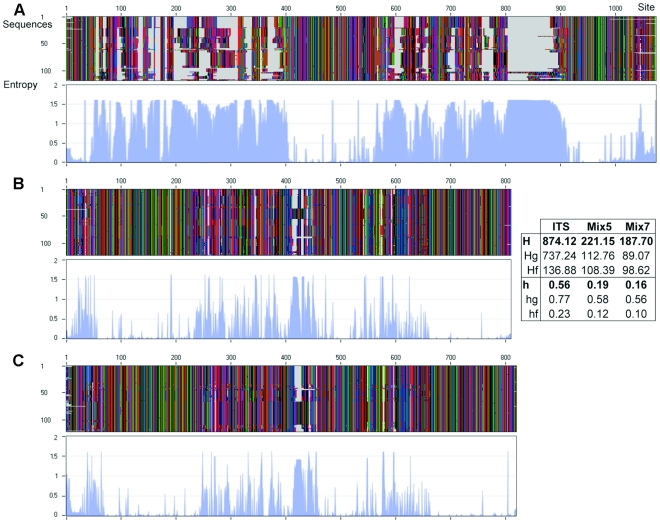
Gaps and entropy (in nats) for each site in the A) ITS, B) Mix5, C) Mix7 T1-0.05 alignments. The values of the cumulative entropy H and relative heterogeneity h are given for the alignment as a whole; subscripted suffixes denote whether the entropy was calculated including (H_g_, h_g_) or excluding gaps (H_f_, h_f_).

Of the three datasets analysed in this work, the cumulative entropy (H = 874.12) and total relative heterogeneity (h = 0.56) were highest for the ITS alignment, for which ca. 59% of all sites contained gaps. Both values were at least three times higher than the corresponding values for the LSU alignments (Figure 10), which had much lower proportions of gap-containing sites (16% for Mix5 and 13% for Mix7). The most homogenous alignment sections within our datasets were the gap-free columns present in both LSU alignments (h_f_ = 0.10 and 0.12). The highest relative heterogeneity scores were those for the gap-containing sites of the ITS alignment (h_g_ = 0.77). Notably, the overall median and average entropy of the LSU alignments were relatively small (0.00 and 0.23, respectively, for Mix7, and 0.04 and 0.27, respectively. for Mix5) but those of the ITS alignments were much higher (0.88 and 0.81).

## Discussion

### The influence of alignment modification and the OTU cutoff value on rarefaction analyses and OTU characteristics

The removal or inclusion of ambiguously aligned regions and the gap treatment had profound effects on the accuracy of phylogenetic analyses. Similar problems have been encountered in previous phylogenetic studies using non-protein-coding sequences, especially for ITS alignments (e.g. [78], [79]) and when dealing with ambiguities (e.g. [80]) or gaps (e.g. [81]). Increases in the kappa value (the ratio of the transition and transversion rates) have been reported to have a positive influence on the distance-based phylogenetic accuracy calculated using the BioNJ algorithm [82]. Different alignments tend to have different kappa values (Table 2) and the kappa values for purines (k_1_) are generally much lower than those for pyrimidines (k_2_). Our results suggest that the Mix7 alignment should yield a higher degree of phylogenetic resolution than either ITS or Mix5. The only indicator of phylogenetic accuracy that can be computed using our data is the number of nodes with high bootstrap support. The values obtained for this parameter were consistent with our expectations: the Mix7 NJ and ME phylogenetic trees had the highest percentage of nodes with the maximum possible bootstrap support value and the highest average bootstrap values when considering only nodes with bootstrap values of 50 or more. This trend was particularly pronounced for the ME trees (data not shown).

MOTHUR uses the furthest neighbour algorithm to define OTUs for rarefaction analysis, whereas FastGroupII relies on the nearest neighbour algorithm. Consequently, the number of OTUs identified for each alignment using MOTHUR was much lower than with FastGroupII, and the MOTHUR analyses were less sensitive to changes in the way the alignments were processed (data not shown). In the MOTHUR analyses, differences in alignment processing had a much less pronounced influence on curve progression than did the sequence cutoff level. This effectively made most of the MOTHUR rarefaction curves congruent. All of the Mix7 rarefaction curves tracked one-another perfectly and only 9 OTUs were detected among the 120 sequences analyzed. Because of its ease of use and widespread adoption, and to avoid unnecessary duplication of labour, all subsequent analyses were conducted on the basis of the OTUs defined using FastGroupII. The relatively large number of OTUs identified using FastGroupII facilitated analysis of the effects of alignment processing. As expected, the cautious deletion of ambiguously aligned characters (as was done in the T3 treatment) consistently reduced the number of OTUs.

The impact of varying the cutoff level on the OTU number was much more apparent for the LSU than for the ITS data. This was attributed to the relatively low level of OTU-defining variation in the LSU alignments. We assume that much of the OTU number stability of the ITS dataset with respect to changes in alignment processing is due to the alignments being somewhat weak to begin with. The Mix5 dataset yielded the highest values for the various diversity indices considered. This is probably due to its relatively high number of singleton OTUs and high degree of species evenness: 71% of its OTUs were singletons, compared to 63% for Mix7. This suggests that when including singleton OTUs, the Mix5 primer pair may be better than Mix7 for assessing fungal biodiversity using LSU rDNA sequences.

Singleton OTUs dominated in all alignments and treatments (Figure 3); this is consistent with empirical findings from previous studies using high-throughput Sanger sequencing e.g. from clone libraries or shotgun sequencing libraries [83].

### Agaricomycotina diversity detected using the ITS and LSU primers

The dominant taxonomic orders identified by analysing the ITS and LSU sequences were very similar, i.e. Agaricales, Russulales, and Boletales (Figure 4). Our results indicate that different sets of primers can be complementary. Remarkably, this was the case for Mix5 and Mix7 even though both pairs target the same region of the LSU gene. Therefore, if a near-complete inventory of the organisms in an environmental sample is desired, the use of “redundant” primers such as the Mix5 and Mix7 pairs should be considered.

The taxonomic affiliations determined by BLAST searching were compared to those derived from phylogenetic trees. This revealed that while the LSU datasets were less diverse than ITS, some OTUs and some of the higher taxa were found only by LSU primers and vice versa. The names assigned to specific OTUs on the basis of BLAST results generally corresponded to monophyletic clades with a high degree of bootstrap support in the ME-K2P analyses (Figures 6, 7, 8). However, the number of monophyletic higher taxa identified in the ITS dataset was comparatively low, presumably because the corresponding alignments were of lower quality (Figure 5). The primary results of the NJ analysis were congruent with the results of the ME analysis; both methods yielded similar phylogenetic patterns for the same datasets. For both methods, the Mix7 alignments had greatest proportion of nodes with the highest possible bootstrap values - 19% as calculated using BioNJ-K2P and 21% as calculated using ME-K2P. The Mix7 dataset also yielded the best results in terms of average bootstrap support for the entire tree when only high values (over 49%) were considered (86% for NJ and 85% for ME method) and in terms of kappa values. The Mix7 dataset thus appears to have the best overall phylogenetic accuracy of the three considered in this work. Compared to the LSU data, the ITS dataset was much more diverse in terms of OTU number but had relatively low bootstrap support.

Overall the higher-level phylogenetic relationships of Agaricomycotina revealed in the LSU and ITS ME phylograms were in agreement with large-scale phylogenies reported by Matheny et al. [84] and Hibbett [11].

### Substitution saturation and entropy

The DAMBE plot of observed numbers of transition and transversion versus divergence may indicate substitution saturation if the increase of transversions exceeds that of transitions with increasing genetic distance [73] due to generally higher frequency of transitional than transversional substitutions in a genome [85]. Many phylogenetic studies (e.g. [86]–[Bibr pone.0032139-Struck1] [88]) have shown that rapidly evolving genes cannot be used for phylogenetic analyses of deep branches because high evolutionary rates lead to multiple substitutions at the same site, saturation of the phylogenetic signal, and incorrect tree reconstruction. Significantly saturated codon positions are often excluded from further analysis (e.g. [89]). Our analyses indicated that despite its relatively small size (ca. 120 sequences) and modest level of K2P divergence, the ITS alignment was extensively saturated in terms of transitions, suggesting a considerable loss of phylogenetic information. This implies that the ITS alignment cannot be used to obtain reliable phylogenetic inferences, a conclusion that was supported by the results of Xia tests [90] conducted using DAMBE. This test, which is based on differently calculated entropies [91], was used as an additional measure of substitution saturation (results not shown). Xia tests were performed for all three alignments, with two tree topologies (symmetrical and unsymmetrical). Owing to recently-implemented improvements in the way DAMBE handles gaps and ambiguities (Xuhua Xia, personal communication, November 1, 2010), three site types were considered: fully resolved sites only; gap-free sites only; and all sites, with gaps treated as unknown states. Highly significant differences (p = 0.00) between the index of substitution saturation (Iss) and the critical Iss (Iss.c) were observed for all alignments and site types other than ITS when examining all sites and treating gaps as unknown nucleotides (in which case p = 0.54). Neither LSU alignment was extensively saturated: in both cases, the Iss was consistently significantly lower than the Iss.c no matter what analytical settings were used. Two more important findings were obtained from the Xia test on the ITS alignment when treating gaps as unknown nucleotides and using an asymmetrical tree topology. First, the difference between Iss and Iss.c became non-significant even when considering only 16 sequences (Iss<Iss.c at p = 0.54), indicating that the ITS alignment is indeed heavily saturated. For 32 sequences, the Iss value was significantly greater than that of Iss.c (Iss>Iss.c at p = 0.00) rendering the ITS alignment useless for phylogenetic analysis. Aside from demonstrating the unsuitability of the ITS alignment for phylogenetic reconstruction, these results also highlight the importance of defining gaps as character states when analysing alignments.

DAMBE only samples 2, 8, 16, or 32 sequences from each alignment. We extrapolated from these results to estimate the theoretical borderline of phylogenetic accuracy for our ITS and LSU alignments (data not shown). A plot of the difference between the two indices (Iss.c-Iss) against the number of sequences tested yielded some surprising results. For fully asymmetrical trees (which were obtained when all sites were analysed with gaps treated as unknown nucleotides), the ITS alignment became unsuitable for phylogeny once 18 or more sequences were included. Conversely, for the LSU alignments, the difference between the Iss.c and Iss values did not reach zero until at least 50 sequences were considered (or 55 in the case of Mix7), once again demonstrating the phylogenetic superiority of LSU data.

Entropy is a more sophisticated measure of sequence variation and is used to quantify the phylogenetic informativeness of alignments. If a given position within an alignment has high entropy, it is informative but this information may be phylogenetically irrelevant or wrong. For example, if some position within an alignment has the highest possible entropy, all possible character states occur at that position with equal frequencies. As such, it will contribute equally well to any phylogenetic partition (disregarding parsimony informativeness or any differential weighting of specific substitutions that may be implemented in models). If the only possible character states for the position are the 4 nucleotides A, C, T and G, maximum entropy could thus support a hard polytomy with four branches. A position has zero entropy if it has the same character state in every sequence in the alignment, making it completely uninformative in phylogenetic terms.

Analyses of the entropy distributions within the ITS and LSU alignments clearly demonstrated that the highest entropy values were associated with gap-containing sites (Figure 10). Most of the highest entropy values occurred in clusters, especially for the ITS dataset. In total, 19 clusters consisting of two or more successive sites with entropy values of more than 1.5 nats were found within the ITS alignment (seven of them were larger than 10 tons). The largest gap-dominated area with entropy values above 1.5 in the ITS alignment was located between alignment positions 804 and 882 (spanning 79 continuous sites) with a mean gap frequency of 91.2% (Figure 8A). Removal or non-consideration of these gaps, as is done by default in FastGroupII and many distance-based phylogenetic analyses, would severely limit the information available from this alignment. Recent studies on alignment performance and accuracy [92], [93] suggest that gaps carry significant but often unexploited tree signals and that there may even be a deterministic relationship between gap content and the level of phylogenetic accuracy (especially for extremely gap-rich and gap-poor alignments), and that the pairwise deletion of gaps (which is common in distance methods used in molecular ecology) often results in saturation-driven losses of phylogenetic signal.

Regions of high entropy were much more dispersed in the two LSU datasets, resulting in relatively thin peaks when entropy values were plotted as a function of alignment position. The Mix5 alignment contained only five clusters featuring two or more successive sites with H>1.5; the longest of these extended over eight sites (412 to 419) with a gap percentage of approximately 77.8% of the nucleotide alignment positions. For Mix7, only one small area (3 ton) with maximal H values was found in the entirety of the alignment (covering positions 577 to 579, for which the gap percentage was 96.9%). The vast majority of the entropy peaks occurred within the D regions of the LSU rDNA gene (Figure 10B, C compared to Figure 1), as might be expected given their universal hypervariability in various kingdoms of the Eukarya (e.g. [44], [46]). The ITS alignment had greater cumulative entropy and total heterogeneity values than either LSU alignment, suggesting that it was either more informative overall or more chaotic.

### Introducing an OTU internal distance area parameter

**Figure 11:**
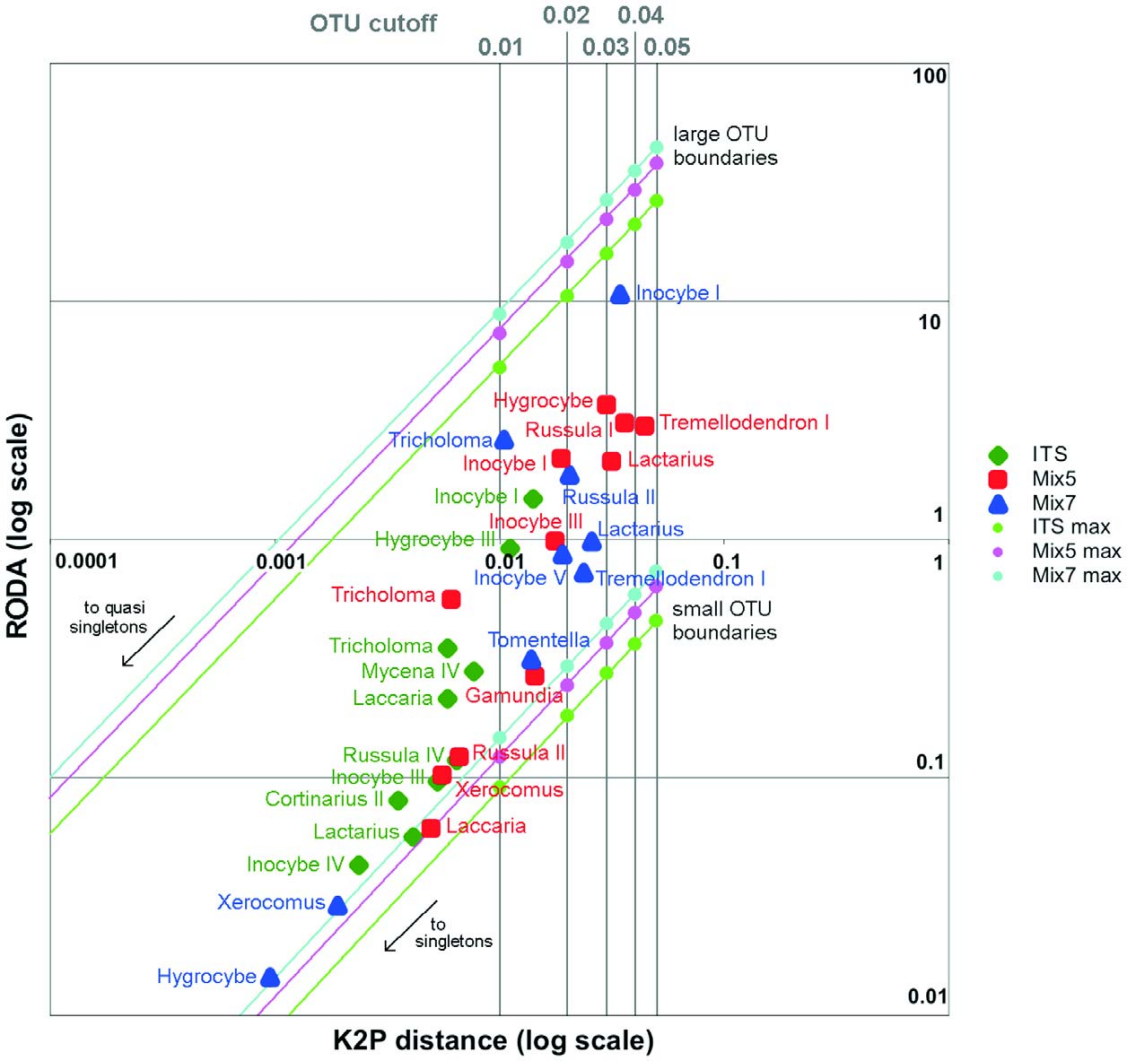
Realized OTU distance area (RODA) vs. K2P distance plot for ITS, Mix5, and Mix7 datasets (T1-0.05 alignments). The OTUs were defined and their internal distances were calculated using FastGroupII. OTU cutoff boundaries were defined in terms of the K2P distance within the OTU.

To describe the effects of changing the cutoff on the number of OTUs identified in an alignment, we created a new type of plot that we have termed an OTU distance area plot (see Figure 11) and some new terminology.

In theory, an OTU can have a positive internal distance only if it contains more than one sequence (and is therefore not a singleton). The “internal distance” of an OTU is hereby defined as the maximum distance between the sequences of the OTU and their last common ancestor node, which is treated as a part of the OTU for this purpose. Quasi-singleton OTUs contain sequences that are identical, but must be differentiated from other doubletons etc., because they have no measurable internal distance, i.e. they act like singletons. To describe the diversity within an OTU, we created the Internal OTU Distance Area (IODA) parameter, which is defined as the product of the internal distance and the number of sequences in the OTU, expressed as a percentage of the tree's maximum possible distance area (which is equal to the number of sequences in the alignment). The Realized OTU Distance Area (RODA) is a related factor that incorporates a correction for the actual genetic distance covered by all OTUs together rather than the maximum possible distance. The minimum possible distance area for an OTU is 2 (since the parameter is not defined for singletons) and the maximum is equal to the total number of sequences in the alignment. The parameter cannot be computed if the cutoff value is 100% because by definition this will yield a situation in which all OTUs consist of (quasi)-singletons. The greater the number of distinct sequences in an OTU, the closer it will be to the top of a distance area plot. On moving leftwards within the plot, the OTUs become increasingly like quasi-singletons, i.e. they have an increasing tendency to contain numerous highly similar sequences. Non-quasi-singleton OTUs with only two sequences will be found on the minimum possible distance area line. The plot can be used to get an overview of the genetic diversity space within a sample in a large environmental genomics study, or to determine the optimal level for defining species and identifying correlations with other data in a systematics study.

In our plots (Figure 11), the ITS tree (Figure 10) contributed 10 OTUs, the Mix5 tree contributed 11, and Mix7 contributed 9. The internal distances (K2P) for all of these OTUs were less than 0.05. Nine of the 30 OTUs analyzed had the highest possible RODA values (all of which were greater than 1); most of these originated from the Mix5 (*Hygrocybe*, *Inocybe* I, *Lactarius*, *Russula* I, and *Tremellodendron* I) and Mix7 (*Inocybe* I, *Russula* II, and *Tricholoma*) datasets, both of which had multiple OTUs containing 11 to 33 sequences. Only one ITS-derived OTU fell into this category (*Inocybe* I, which contained 41 sequences). The maximum RODA value was different for each dataset but was always achieved by the most sequence-rich OTUs. The plot indicated that the Mix7 dataset was the most heterogeneous, with RODA values ranging from 10.68 for *Inocybe* I to 0.01 for *Hygrocybe* and 0.02 for *Xerocomus*, both of which were located on the minimum possible distance area line. The Mix5 OTU pool was very sensitive to the K2P distance: even going from 0.05 to 0.04 resulted in the exclusion of one OTU (*Tremellodendron* I). The LSU OTUs were distributed across the entire area of the plot. In contrast, the ITS OTUs clustered together and none extended beyond the line corresponding to a K2P distance of 0.02. This suggests that relatively low OTU cutoffs should be used when analyzing ITS data in ecological studies.

OTU distance area plots could also be used to monitor the behaviour of individual sequences or groups of sequences in response to changes in the cutoff value, for example by constructing a plot showing all the OTUs obtained at different cutoff values and then drawing lines to connect the OTUs obtained with the higher cutoffs and their derived “children” that are obtained with more stringent cutoffs. This would make it easy to see how OTUs split (or merge) as cutoffs change and to identify OTUs that are robust to changes in the cutoff value. However, it would be necessary to design and develop new software tools to monitor OTU behaviour in this way for situations where changing the cutoff changes the maximum possible IODA value.

### Conclusions

We have developed simple methods and parameters that can be used to assess the quality of the results obtained with different primer pairs, OTU cutoff values, and alignment treatments when conducting large-scale molecular ecology studies on systems whose true evolutionary history is unknown and for which sampling and sequencing errors cannot be ruled out. We focused on tools for exploring data to determine whether bifurcating phylogenetic trees provide realistic representations of diversity for large-scale molecular ecology. It was assumed that a “correct” alignment would accurately reflect the homology of nucleotide positions and that the methods and models of phylogenetic inference would translate this into a bifurcating phylogenetic tree that could be used to analyze the closeness of relationships within the sample. Quality parameters are needed in order to determine whether alignments and phylogenetic trees accurately reflect the diversity within the sample; one such parameter is the entropy-based relative heterogeneity. Phylogenetic inaccuracies can arise when examining very heterogeneous alignments. The creation of alignments is dependent on the availability of some kind of reference point, i.e. a series of homogenous columns with low entropy. However, once the alignment has been created, it is possible that these reference columns could be cropped without reducing the homological value of the remaining character columns. The heterogeneity of an alignment is largely confined to blocks that typically vary in length, contain numerous gaps, and have many and uncertain positions due to their high nucleotide heterogeneity. An alignment's relative heterogeneity score is thus a simple indicator of its phylogenetic usefulness; ideally, it should be low but non-zero. The heterogeneity score can take values between 0 and 1; it will be necessary to perform simulations with a range of large datasets to determine the optimal range for phylogenetic studies.

Overall, our results suggest that for the moment, the ITS and LSU primers should be used together but that when analyzing raw ITS sequence diversity, one should rely on local and not global alignment data and sequence identification based on BLAST searches. Great care should be taken when defining OTUs and constructing phylogenetic trees on the basis of ITS data, as splits tree approaches [94], the Xia test [90], the Shannon Heterogeneity In Alignments Tool [95], and the new parameters described in this paper would indicate them as not useful. One alternative to their use is to simply refrain from attempting phylogenetic tree reconstruction altogether. Another potentially viable approach would be to assemble subtrees on a guiding backbone tree or to first attempt to find the sequences that cause most of the conflict and unresolvedness e.g. by using approaches based on taxon resampling in conjunction with optimized indices of phylogenetic resolution [96]. While the latter of these two options suffers from a lack of software tools and might be computationally prohibitive, it offers the potential for creating a tree using only a subset of the available data and grafting additional sequences onto it as subtrees or as lists of sequences closely allied by BLAST and pairwise distances.

It is hard to identify an optimal OTU cutoff value if the OTUs are defined in terms of distances that themselves depend on potentially faulty alignments. In the absence of reliable automated methods, it is necessary to rely on taxonomic expertise and an understanding of the species being studied and their sequences when defining appropriate cutoff levels for specific groups of organisms. To this end, it would be useful to develop new software to investigate and visualize the behaviour of OTUs or taxa as a function of their internal distances, using methods such as the OTU distance area plots discussed in this paper.

## Supporting Information

File S1 **Perl script for condensing BLAST output.**
(TXT)

File S2 **Simulation of sampling biases.**
(DOC)

Table S1 Fungal strains used to test the specificity of the new 28S rDNA primers and the results of the specificity tests.
(DOC)

Figure S1 **Resampling profile of 120 of 120 000 items (A, B, C, D) picked over 100 replicates.** Vertical lines correspond to 10 items. Average percentage and S = standard deviations on average percentage are given on the bottom.
(TIF)
