# Supplementary material for: Diversity Measures in Environmental Sequences Are Highly Dependent on Alignment Quality—Data from ITS and New LSU Primers Targeting Basidiomycetes
Source: PLoS One. 2012 Feb 21;7(2):e32139. doi: 10.1371/journal.pone.0032139 (PMC3283731; doi:10.1371/journal.pone.0032139)
Supplement: File S2 — Simulation of sampling biases. (DOC) [file pone.0032139.s002.doc]

**File S2.** Simulation of sampling biases

In order to assess how many sequences to collect without overly creating additional bias, we assumed that a PCR clone library might contain e.g. 120 000 clones that were derived from unbiased PCR from soil, and unbiased cloning, and contained equally distributed distinct identities. We then simulated the picking of clones by a resampling procedure as follows:

First we created a random list of clone identities (which may be membership to a taxonomic group or OTU). We used http://www.daimi.au.dk/~biopv/php/fabox/random_sequence_generator.php (last accessed June 3, 2011) for this process, specifying 120 000 nucleotides as sequence length, and 0.25% base frequencies for A, C, T, and G. A, C , T and G were then replaced by items A, B, C and D standing for four distinct clone identities. A random number calculator (http://www.random.org/integers/?mode=advanced, last accessed June 3, 2011) was then used 100 times to generate 120 random numbers between 1 and 120 000 each time. By the aid of perl scripts and Excel we then picked the appropriate item from the random list and counted the resulting A, B, C and D items per bootstrap replicate. Doing so, we found the average item frequency ranged between 24.3 and 25.5 % with only small standard deviations. This approach might generally be useful for deciding on sample numbers where there is prior information on expected diversity.
