# Supplementary material for: Diversity Measures in Environmental Sequences Are Highly Dependent on Alignment Quality—Data from ITS and New LSU Primers Targeting Basidiomycetes
Source: PLoS One. 2012 Feb 21;7(2):e32139. doi: 10.1371/journal.pone.0032139 (PMC3283731; doi:10.1371/journal.pone.0032139)
Supplement: Table S1 — Fungal strains used to test the specificity of the new 28S rDNA primers and the results of the specificity tests. (DOC) [file pone.0032139.s003.doc]

**Table S1.** Fungal strains used for specificity test of new 28S rDNA primers and results.

| Species | Strain* | Order | Division | Source / Collector  (geographical origin)** | Ecology*** | PCR amplicon with primer pair  Mix5 Mix7 | |
| --- | --- | --- | --- | --- | --- | --- | --- |
| *Agaricus silvaticus* | CBS 245.51 | Agaricales | Basidiomycota | CBS (Netherlands) | S | + | + |
| *Baeospora myosura* | SE 194 | Agaricales | Basidiomycota | Susanne Theuerl (Germany) | S | + | + |
| *Bjerkandera adusta* | DSM 3375 | Polyporales | Basidiomycota | DSMZ (Germany) | WD | + | + |
| *Coprinopsis cinerea* Okayama 7 | FGSC 9003 | Agaricales | Basidiomycota | David Moore (Japan) | S | + | + |
| *Daedaleopsis confragosa* | SE 140 | Polyporales | Basidiomycota | Danuta Kapturska (Germany) | WD | + | + |
| *Dichomitus squalens* | DSM 9615 | Polyporales | Basidiomycota | DSMZ (Germany) | WD | + | + |
| *Hygrophoropsis aurantiaca* | DSM 4518 | Boletales | Basidiomycota | DSMZ (Germany) | EM | + | + |
| *Laccaria bicolor* | S238N | Agaricales | Basidiomycota | INRA (France) | EM/S | + | + |
| *Lycoperdon perlatum* | DSM 8678 | Agaricales | Basidiomycota | DSMZ (Germany) | S | + | + |
| *Marasmius androsaceus* | CBS 241.53 | Agaricales | Basidiomycota | CBS (France) | LD/WD | + | + |
| *Micromphale perforans* | CBS 209.47 | Agaricales | Basidiomycota | CBS (France) | LD | + | + |
| *Morchella conica* | DSM 10464 | Pezizales | Ascomycota | DSMZ (Switzerland) | S | - | - |
| *Morganella pyriformis* | DSM 8676 | Agaricales | Basidiomycota | DSMZ (Germany) | WD | + | + |
| *Mucor fragilis* | SE 63 | Mucorales | Zygomycota | Verena Blanke (Germany) | S | - | - |
| *Mucor hiemalis* | SE 64 | Mucorales | Zygomycota | Carsten Renker (Germany) | S | - | - |
| *Paxillus involutus* | SE 198 | Boletales | Basidiomycota | Patricia Luis (Germany) | EM | + | + |
| *Phanerochaete chrysosporium* | DSM 1556 | Polyporales | Basidiomycota | DSMZ (USA) | WD | + | + |
| *Phlebia radiata* | DSM 5111 | Polyporales | Basidiomycota | DSMZ (Germany) | WD | + | + |
| *Polyporus squamosus* | CulTenn 7245-1 | Polyporales | Basidiomycota | SMF (Sweden) | WD | + | + |
| *Postia stiptica* | SE 191 | Polyporales | Basidiomycota | Susanne Theuerl (Germany) | WD | + | + |
| *Stereum hirsutum* | DSM 3281 | Russulales | Basidiomycota | DSMZ (Germany) | WD | + | + |
| *Stereum sanguinolentum* | DSM 3282 | Russulales | Basidiomycota | DSMZ (Germany) | WD | + | + |
| *Verpa conica* | VEJ3M | Pezizales | Ascomycota | Harald Kellner (Germany) | S | - | - |
| *Xylaria longipes* | SE 258 | Xylariales | Ascomycota | Danuta Kapturska (Germany) | WD | - | - |

*CBS = Centraalbureau voor Schimmelcultures (Utrecht), CulTenn = internal strain collection of the Ecology and Evolutionary Biology Department at the University of Tennessee (Knoxville), FGSC = Fungal Genetics Stock Center (Kansas City), SE = internal strain collection of the Soil Ecology Department at the Helmholtz Centre for Environmental Research (Halle); **DSMZ = German Collection of Microorganisms (Braunschweig), INRA = Institut National de la Recherche Agronomique (Paris), SMF = Swedish Mycological Society; ***S = saprotrophic, WD = wood decaying, EM = ectomycorrhizal, LD = litter dwelling; fungal strains were cultivated on standard MEA agar (15 g malt extract, 20 g agar, per 1L distilled water) for 4 weeks at room temperature with two exceptions: *H. aurantiaca* strain was grown on original BAF medium (DSMZ medium no. 392) and *Mucor* strains were grown in liquid medium (15 g glucose, 5 g malt extract, 0.5 g KH_2_PO_4_, 0.5 g MgSO_4_.x 7H_2_0, and 0.05 g CaCl_2_x2H_2_O, per 1L distilled water) for 5 days at room temperature until harvesting; + = amplified, - = not amplified.
