# Supplementary figures and images for: Diversity Measures in Environmental Sequences Are Highly Dependent on Alignment Quality—Data from ITS and New LSU Primers Targeting Basidiomycetes
Source: PLoS One. 2012 Feb 21;7(2):e32139. doi: 10.1371/journal.pone.0032139 (PMC3283731; doi:10.1371/journal.pone.0032139)

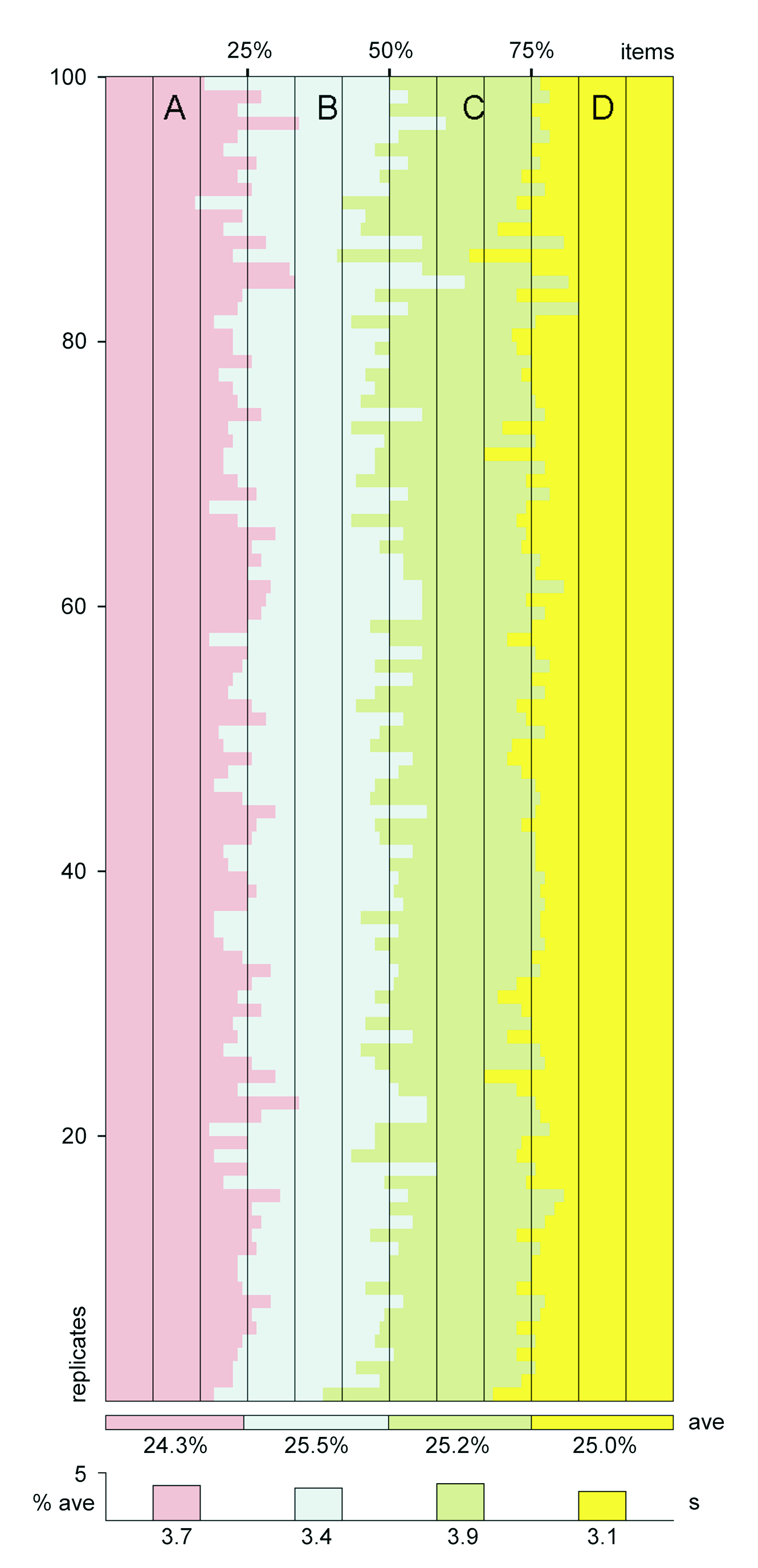

Supplement: Figure S1 — Resampling profile of 120 of 120 000 items (A, B, C, D) picked over 100 replicates. Vertical lines correspond to 10 items. Average percentage and S = standard deviations on average percentage are given on the bottom. (TIF) [file pone.0032139.s004.tif]
